# Norovirus Epidemiology in Africa: A Review

**DOI:** 10.1371/journal.pone.0146280

**Published:** 2016-04-26

**Authors:** Janet Mans, George E. Armah, A. Duncan Steele, Maureen B. Taylor

**Affiliations:** 1 Department of Medical Virology, University of Pretoria, Pretoria, South Africa; 2 Noguchi Memorial Institute for Medical Research, University of Ghana, Legon, Ghana; 3 MRC Diarrhoeal Pathogens Research Unit, University of Limpopo, Pretoria, South Africa; University of Parma, ITALY

## Abstract

Norovirus (NoV) is recognised as a leading cause of gastroenteritis worldwide across all age groups. The prevalence and diversity of NoVs in many African countries is still unknown, although early sero-prevalence studies indicated widespread early infection. Reports on NoVs in Africa vary widely in terms of study duration, population groups and size, inclusion of asymptomatic controls, as well as genotyping information. This review provides an estimate of NoV prevalence and distribution of genotypes of NoVs in Africa. Inclusion criteria for the review were study duration of at least 6 months, population size of >50 and diagnosis by RT-PCR. As regions used for genotyping varied, or genotyping was not always performed, this was not considered as an inclusion criteria. A literature search containing the terms norovirus+Africa yielded 74 publications. Of these 19 studies from 14 out of the 54 countries in Africa met the inclusion criteria. Data from studies not meeting the inclusion criteria, based on sample size or short duration, were included as discussion points. The majority of studies published focused on children, under five years of age, hospitalised with acute gastroenteritis. The mean overall prevalence was 13.5% (range 0.8–25.5%) in children with gastroenteritis and 9.7% (range 7–31%) in asymptomatic controls, where tested. NoV GII.4 was the predominant genotype identified in most of the studies that presented genotyping data. Other prevalent genotypes detected included GII.3 and GII.6. In conclusion, NoV is a common pathogen in children with diarrhoea in Africa, with considerable carriage in asymptomatic children. There is however, a paucity of data on NoV infection in adults.

## Introduction

Norovirus (NoV) is well established as a major cause of viral gastroenteritis across all age groups [1]. These small, non-enveloped single-stranded RNA viruses cause self-limiting disease in healthy individuals within 10–51 hours after exposure. Symptoms include nausea, vomiting, watery diarrhoea, stomach cramps, headache and fever [2]. Children, the elderly and immune-compromised persons may experience more severe symptoms and/or extended duration of illness or even chronic diarrhoea. No antivirals are presently available to treat NoV infection [3] and vaccine development is still in the clinical trial phase [4]. With the introduction of the rotavirus (RV) vaccine in many parts of the world NoV is becoming the leading cause of viral gastroenteritis in young children [5, 6].

Norovirus strains are classified into seven genogroups (G), based on the analysis of the complete amino acid sequence of the capsid protein. GI, GII and GIV are associated with disease in humans and within each genogroup there are multiple genotypes [7, 8]. Although NoVs are characterised by high genetic diversity and recombination, one genotype, GII.4, causes >80% of NoV gastroenteritis outbreaks and the global predominance of GII.4 has been observed over the past 20 years. A new NoV GII.4 variant emerges every few years, rapidly replacing the circulating dominant strain, and spreading globally to become the dominant cause of NoV gastroenteritis [9].

Significant advances in the understanding of the evolution and diversity of NoVs and the global nature of NoV gastroenteritis have been made in the last decade [10–12]. Successful NoV surveillance systems have been implemented in Europe, the United States, Oceania and Asia leading to a dramatic increase in information on NoV prevalence and diversity. In contrast, there are very few NoV surveillance efforts on the African continent. Early sero-prevalence studies performed in southern Africa indicated widespread NoV infection at a young age [13, 14]. More recently a growing number of reports have described NoV infections in various countries in North and Sub-Saharan Africa. This review summarises the data from investigations with varying study design to provide an estimate of overall prevalence and distribution of genotypes of NoVs in Africa, to identify crucial data gaps and to discuss approaches to further NoV research in Africa.

## Methodology

A literature search on PubMed containing the terms norovirus+Africa yielded 74 publications (3 August 2015). Studies which had a duration of at least 6 months, a study population size of >50 and used NoV-specific RT-PCR detection were included. Nineteen investigations from 14 of the 54 countries in Africa met the inclusion criteria. The overall prevalence was calculated from these 19 studies. Data from additional studies which did not meet the inclusion criteria but provided genotyping data were included in the analysis of NoV diversity. Available NoV nucleotide sequence data from any African country on GenBank were analysed with the Online NoV Genotyping Tool (http://www.rivm.nl/mpf/norovirus/typingtool) to assign genotypes and GII.4 variants [15]. GenBank accession numbers—Botswana: GQ166482-GQ166527; Burkina Faso: KF434302-KF434317, JX416387-JX416419; Cameroon: JF802497-JF802511, KJ946387-KJ946411; Central African Republic: JN699050; Djibouti: EF190918-EF190920; Egypt: EU876882-EU876889, EU876892-EU879893; Ethiopia: JF909061-JF909043, KM589642-KM589661; Ghana: DQ013131-DQ031143, KF275021-KF275025, EU315735-EU315748, KC865052; Kenya: KF793788-KF793798, KF279373-KF279391, KF916584-KF916585, KF808211-KF808254; Libya: HQ919943-HQ919917; Morocco: KJ162360-KF162391, KJ735099, JX645691-JX645694, KJ404069, JF803959; Malawi: AB234229-234215; Nigeria: JN871684; Senegal: JN699035; South Africa: GU138767-GU138772, HQ008054-HQ008504, HQ201641-HQ201679, JN191355-JN191381, KC495619-KC495687, KR904207-KR904808; Tanzania: KJ394491-KJ394507, KJ862463-KJ862516; Tunisia: JQ692877-JQ692937, EU916955-EU916961, EU650205-EU650227, JN418482-JN418492, JX455837-JX455896, FJ905454-FJ905458, FJ905400-FJ905406, JX401281; Uganda: JN699046, JN989560.

## Norovirus Prevalence and Age Distribution

**Fig 1:**
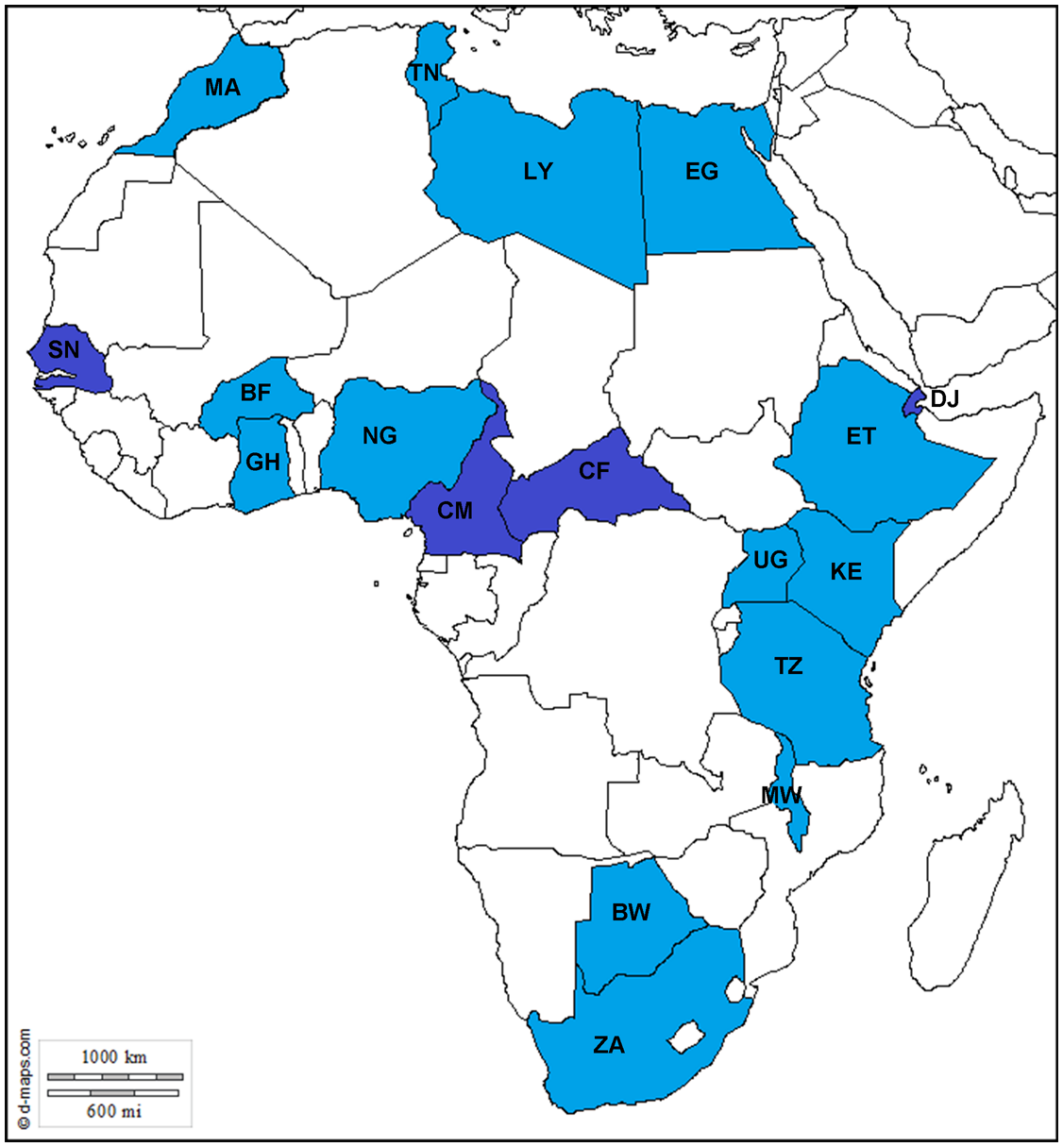
Map of Africa indicating the countries from which prevalence and diversity data was obtained (light blue) and countries where only NoV genotype data was available (dark blue). BF—Burkina Faso, BW—Botswana, CF—Central African Republic, CM—Cameroon, DJ—Djibouti, EG—Egypt, ET—Ethiopia, GH—Ghana, KE—Kenia, LY—Libya, MA—Morocco, MW—Malawi, NG—Nigeria, SN—Senegal, TN—Tunisia, TZ—Tanzania, ZA—South Africa. Reprinted from d-maps.com under a CC BY license, with permission from Daniel Dalet, original copyright 2007–2015 (http://d-maps.com/carte.php?num_car=737&lang=en).

The majority of the studies focused on children seeking medical care for gastroenteritis symptoms (hospitalised and/or outpatients). The overall NoV prevalence in individuals with gastroenteritis from 19 studies originating from 14 African countries (Fig 1) was 13.5% (961/7141, 95% CI 12.7–14.3). This prevalence correlates with the 14% (CI 11–16) reported in a high-mortality developing setting [1]. Ahmed and colleagues suggest that the lower NoV prevalence in this setting, compared to 20% (CI 17–22) in developed countries, does not reflect a lower NoV burden but rather a wider diversity and higher prevalence of other gastroenteritis pathogens [1]. Six of the 19 studies reported data on asymptomatic controls with an overall prevalence of 9.7% (181/1868, 95% CI 8.4–11.1) (Table 1). The NoV prevalence in asymptomatic patients estimated by Ahmed and co-workers [1] from 20 studies across the world was 7% (CI 3–10). This discrepancy is likely due to the small number of studies (4/20) from high-mortality developing countries included in their analysis [1].

**Table 1:** Summary of NoV prevalence and diversity data from 19 studies conducted in 1976–1979, and between 1997 and 2013 in 14 African countries.

Country	Study duration	Population		Study setting	NoV prevalence (NoV^+^/total)		Median age (months) [IQR], (range) of symptomatic NoV population	Peak NoV detection	Genotypes (Predominant type highlighted)	GII.4 variants	Reference
		Age range	Size		Symptomatic	Asymptomatic					
Botswana	7 years 2000–2006	Children	100	H	22% (16/74)	31% (8/26)	NR	NR	GI.P1, **GII.P4,** GII.P7, **GII.4**, GII.20	Farmington Hills 2002, Hunter 2004, Yerseke 2006a, New Orleans 2009, unassigned GII.4	[19]
Burkina Faso	11 months 2009–2010	<5 years	309	H	12% (37/309)	-	NR	Dec-Feb (Cool, dry season)	GI.1, GI.3, GI.6, GI.7, GI.NA* GII.1, **GII.4**, GII.6, GII.7, GII.8, GII.10, GII.14, GII.16, GII.17	Sydney 2012, unassigned GII.4	[20]
Egypt	12 months 2006–2007	1 month– 18 years	230	H	13.4% (31/230)	-	NR	Oct-May (Cold season)	GI.1, GI.3, GI.4, GI.5, GI.9, GII.P3, **GII.P4,** GII.P21/GII.3, **GII.4**, GII.15	Yerseke 2006a, Den Haag 2006b, Osaka 2007	[21]
Ethiopia	6 months 2008–2009	<5 years	257 (152-IP) (48-OP)	H	8% (16/200)	7% (4/57)	10.5 (4.9) ^#^	NR	GI.3, GI.5, GI.6, GII.2, **GII.3**, GII.4, GII.6, GII.12, GII.14, GII.17	unassigned GII.4	[22]
Ghana	2 years 1998–2000	≤2 years	82	H	15.9% (13/82)	-	NR	Dec-Apr (Cool, dry season)	GI.P1, GI.P5, GII.2, **GII.4**, GII.P8/GII.14	Osaka 2007, unassigned GII.4	[23]
Ghana	1 year 2007–2008	≤13 years	1234	H	16.6% (91/548)	7.0% (48/651)	13 [8–22] (cases)40 [20–82] (controls)	NR	No genotyping performed	-	[24]
Kenya	16 months 1999–2000	<14 years	105	C	18.9% (7/37)	13.2% (9/68)	NR	NR	GI.3, GII.2, GII.4, GII.6, GII.12, GII.14, GII.17	unassigned GII.4	[25]
Libya	1 year 2007–2008	<5 years	520	H	17.5% (91/520) 13.2% (36/260 IP) 21.2% (55/260 OP)	-	9 [6–12]	May-Aug (Summer) Peak in Aug	GI.5, **GII.4**	Yerseke 2006a, Den Haag 2006b, unassigned GII.4	[26]
Malawi	1 year 1998–1999	<5 years	398	H	6.5% (26/398)	-	6 (1–28)	March (Rainy season)	**GII.P3**, GII.P4, GII.P12, GII.NA	unassigned GII.P4	[27]
Malawi	10 years 1997–2007	<5 years	2446	H	11.3% (220/1941)	11.8% (60/505)	6 (<1–48)	Aug-Nov (End of dry season) and Feb-Mar (End of rainy season)	GI.3, GI.5, GI.7, GI.9,*GI*.*11*, *GI*.*14*^$^ GII.2, GII.3, **GII.4**, GII.6, GII.7, GII.10, GII.11, GII.12, GII.13, GII.15, GII.16, GII.P4/GI.5	Camberwell 1994, US95_96, Farmington Hills 2002, Houston 2002, Hunter 2004 Yerseke 2006a, Den Haag 2006b, Apeldoorn 2007	[28]
Morocco	1 year 2011	<5 years	335	H	16.1% (54/335)	-	14 (2–48)	June (Summer)	GII.3, **GII.4**, GII.13, GII.16, GII.17	New Orleans 2009, Sydney 2012, unassigned GII.4	[29]
Morocco	13 months 2011–2012	>2 months < 5 years	122	H	0.8% 1/122^+^	-	-	-	No genotyping performed	-	[30]
Nigeria	8 months 2010–2011	≤5 years	55	C	25.5% (14/55)	-	NR	Peak in October (Dry season)	GI.NA, GII.4	-	[31]
South Africa	12 months 2008	≤13 years	245	H	14.3% (35/245)	-	8 (2–24)	Oct-Dec (Spring, early summer)	GI.2, GI.7, GI.8, GII.1, **GII.4**, GII.6, GII.7, GII.10, GII.13, GII.14, GII.16	Hunter 2004, Yerseke 2006a, New Orleans 2009, unassigned GII.4	[32]
Tanzania	1 year 2010–2011	<2 years	1266	H	18.3% (129/705)	9.3% (52/561)	9 (cases) 12 (controls)	April (End of wet season)	GI.5, GI.7, GII.Pg, GII.Pe, **GII.P4**, GII.P13, GII.P16, GII.P21	New Orleans 2009, unassigned GII.4	[33]
Tunisia	1976–1979 Archival WHO Global Study	<14 years	92	H	10.9% (10/92)	-	NR	NR	GI.3, GII.NA, GII.2, GII.3	-	[34]
Tunisia	4 years 2003–2007	<12 years	788	H+C	16.2% (128/788)	-	NR	No clear pattern	GI.P2, GI.P4, **GII.P4**, GII.P8, GII.P21, GII.1, GII.2, GII.3, **GII.4**, GII.8, GII.14	US95_96, Hunter 2004	[35]
Tunisia	3 years 2007–2010	<13 years	407	H	9.3% (38/407)	-	NR	No seasonal peak	GI.Pb/GI.6, GI.2, GII.4, **GII.P21/GII.3**, GII.6, GII.7/GII.6, GII.8	Hunter 2004, Den Haag 2006b, Osaka 2007, unassigned GII.4	[36]
Uganda	1976–1979 Archival WHO Global Study	<14 years^	53	H	7.5% (4/53)	-	NR	NR	GI.NA, GI.5, GII.6, GIV.1	-	[34]

AS—asymptomatic, C—specimens collected in the community, H-hospitalised children as well as children seeking medical care at clinics, IP-inpatient, OP-outpatient, NR- not reported

*- GI.NA or GII.NA—strains that are not assigned to any recognised genotype by the Online NoV Genotyping Tool

^+^—single round RT-PCR detection—could explain very low detection rate

^- One NoV-positive specimen (GI.5) was obtained from an adult involved in specimen collection in the study

^$^- Designated as GI.11 and GI.14 in [28], sequences are not available on GenBank for analysis with NoV Genotyping Tool

^#^-Mean age and standard deviation

The ages of the study populations varied from ≤2 years (2 studies), ≤5 years (8 studies), ≤12 to ≤14 years (8 studies) and up to 18 years (1 study). The NoV prevalence in children ≤2 years was significantly higher (18%) than in studies which included children up to 5 years of age (11.8%) (chi square test, p<0.001). Several studies only reported the mean or median age of the NoV-positive population and these ranged from 6 to 14 months (Table 1). These data suggest that children under 5 years of age, and especially children younger than 12 months, are very likely to experience NoV infection. This observation is not unique to developing African countries. Several other studies in children hospitalised with gastroenteritis have observed that NoV infections are most often detected in children <2 years of age [16–18].

It is well described that NoVs are also often detected in asymptomatic individuals [37]. Compared to a recent global estimate of the prevalence of NoV infection in symptomatic and asymptomatic individuals of 20% and 7% respectively [1], the difference observed between the prevalence in symptomatic and healthy children is much smaller in the African setting (13.5% vs 9.7%), although it is statistically significant (chi square test, p<0.001). This could be due to various factors which include the definition of asymptomatic or healthy controls. Only one study in Malawi [28] and the study in Tanzania [33] used a defined diarrhoea-free period (2 or 4 weeks) prior to control specimen collection. In the other four studies which included controls, non-diarrhoeal specimens were collected without any definition of a diarrhoea-free period prior to specimen collection. A proportion of the infections detected in asymptomatic patients could therefore represent virus shedding from a recent infection, since it has been reported that NoVs can be shed for up to three weeks after infection [38, 39]. In developing countries children might be exposed to NoV more frequently than in more developed settings, leading to repeated infections which could be asymptomatic. The studies included in this review varied a lot in terms of reporting of co-infections with other enteropathogens. There was no general trend, although 8/19 studies reported NoV/RV co-infections.

The Global Enteric Multicenter Study (GEMS) evaluated the burden and aetiology of moderate-to-severe diarrhoea at seven sites in sub-Saharan Africa (Kenya, Mali, Mozambique, The Gambia) and South Asia (Bangladesh, India, Pakistan) over a three year period [40]. Based on adjusted attributable fraction (AF) calculations NoV was significantly associated with moderate-to-severe diarrhoea only in The Gambia (AF 8.9 (4.3–13.4)). This reflects the high rate of asymptomatic NoV infections at most of the GEMS sites. In the GEMS study asymptomatic controls were matched in terms of age, sex and area of residence and these controls had to be diarrhoea-free for 7 days before enrolment. This is however a very short diarrhoea-free period and the GEMS study therefore likely underestimated the burden of NoV diarrhoea. The symptomatic or asymptomatic outcome of a NoV infection is possibly a consequence of many factors e.g. the immune status of the individual, previous exposure to NoV, genotype and co-infecting pathogens to name a few. Therefore NoV cannot be disregarded as an important diarrhoeal pathogen based on high detection rates in asymptomatic individuals.

Using a different approach Platts-Mills and colleagues [41] investigated the pathogen specific burden of community diarrhoea in children (0–2 years) in a multisite birth cohort study (MAL-ED) spanning eight sites in Africa (South Africa (SA), Tanzania), Asia (Bangladesh, India, Nepal, Pakistan) and South America (Brazil, Peru). Overall the AF for NoV GII was 5.2% (3.0–7.1) for children in their first year of life and 5.4% (2.1–7.8) in the second year of life. The prevalence of NoV GII in diarrhoeal specimens in the second year of life was 31.5% and 17.7% in non-diarrhoeal specimens at the Venda site in SA. This is considerably higher than the NoV prevalence in children hospitalised with gastroenteritis in the rest of SA [32]. This could indicate a substantially higher NoV burden linked to less severe gastroenteritis symptoms in the SA community. The burden of NoV diarrhoea peaked during 6–12 months of age in Venda, in agreement with the Africa studies reviewed here. The MAL-ED study underlines NoV as an important pathogen causing a significant burden of diarrhoeal disease at the community level.

Supporting data of the importance of NoV in Africa comes from the Phase III, randomized, placebo-controlled efficacy clinical trial of the human RV vaccine (Rotarix, GSK Biologicals, Rixensart) in Malawi and SA [42]. In the moderate-to-severe diarrhoea cases identified in the Malawian subjects, NoV was detected as a single pathogenic agent by real-time PCR in 114/751 (15%) of faecal specimens collected from infants with diarrhoea under one year of age. An additional 18/751 (2%) of specimens contained NoV plus another virus (RV [n = 7], astrovirus [n = 6] and sapovirus [n = 5]). Of the 132 NoVs identified as either single or mixed infections, the majority were GII (88.6%) with only 8% being GI (Nigel Cunliffe, personal communication). A total of 408 diarrhoeal specimens collected during the study in South African infants were screened for NoV using real-time reverse transcriptase PCR [38]. Noroviruses were detected in 13% (53/408) of cases, comprising 88.7% GII and 13.2% GI strains. Mixed infections were common with NoVs detected in combination with RV [n = 21], adenovirus [n = 7], sapovirus [n = 5] and astrovirus [n = 4]. In fact, NoV GII strains were the only enteric virus detected in 19 cases and GI in 4 cases of moderate-to-severe diarrhoea during the study period (Nicola Page, personal communication).

## Norovirus Seasonality

The studies analysed in this review represent North Africa (4), West Africa (3), East Africa (4) and southern Africa (3) (Fig 1). Compared to the northern hemisphere where NoV disease peaks in the winter season, NoV seasonality is less obvious in the African region. In North Africa both Libya and Morocco reported peak NoV detection in the summer months (June-September) however, in Tunisia NoV infections were detected throughout the year with no clear seasonal pattern and in Egypt the NoV infections peaked in the cold season (October—May) (Table 1). In West Africa (Burkina Faso and Ghana), NoVs were detected most often in the cool dry season (December to February/April), however the study from Nigeria reported a peak at the end of the rainy season (October). In East Africa, only the study from Tanzania reported seasonal data and NoV infections were most frequently detected at the end of the rainy season (April). In Southern Africa, a peak in NoV infections was observed in SA in the spring/early summer time (September-November) and during/at the end of the rainy season in Malawi (Table 1). In Egypt and Ghana the NoV season overlapped with the RV season, both taking place in the cool, dry season. However, the peak NoV and RV detection periods did not overlap for the rest of the studies.

Climate regions on the African continent can be divided into the tropics, where a wet and dry season occur, and Northern and Southern Africa which experience four seasons, albeit not with the same defined boundaries as temperate countries in the Northern hemisphere. One would expect similar seasonal patterns in the regions with similar climates, however based on the studies reviewed here no pattern is evident. Factors that could influence norovirus seasonality include temperature, humidity, rainfall, population density and human behaviour. Studies on murine NoV have shown that low absolute humidity is favourable for virus survival [43]. Several countries in Africa observed a peak in NoV activity in the cooler, dry months of the year. This might be related to better virus survival under low humidity conditions and possible crowding due to lower temperatures. Varying population densities and diverse cultural practices could account for the differences in seasonality that is observed across the continent. In addition the high level of asymptomatic NoV infection could contribute to less well defined seasonal patterns because of frequent exposure to virus unrelated to climate conditions.

## Norovirus Genotype Diversity and Recombinants

**Fig 2:**
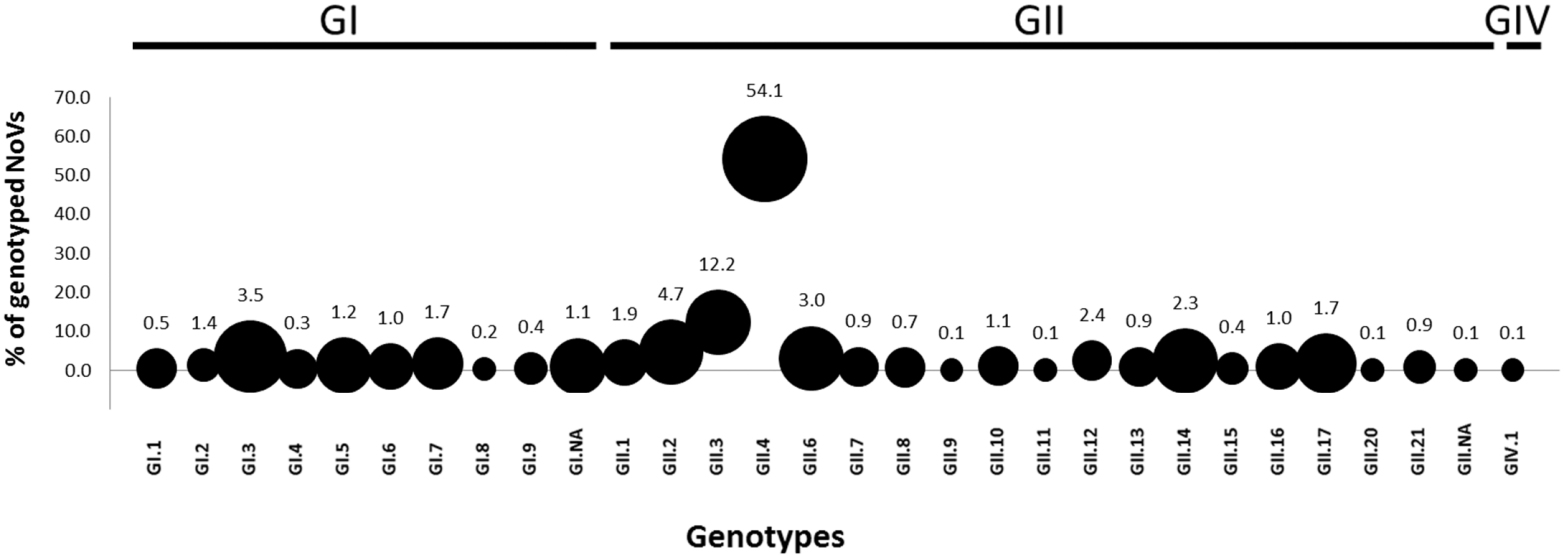
Prevalence of various NoV GI, GII and GIV capsid-based genotypes detected in children with gastroenteritis in 18 African countries between 1976–1979 and 1997–2013. A total of 940 NoV strains were genotyped, the percentage of each genotype out of the total typed strains is indicated above each circle. The size of the circle represents the geographical distribution of a given genotype. The smallest circle represents 1/18 countries and the largest circle represents 14/18 countries.

Genotype data from 18 African countries (Fig 1) were analysed to determine the most prevalent and widely distributed NoV genotypes. Data from 17 studies that met the inclusion criteria for prevalence calculation (Table 1), four studies that did not meet the inclusion criteria (Table 2) and the available NoV sequence data on GenBank originating from Africa were combined. Genogroup II strains represented 84.1% (range 71–100) of all detected NoVs and GI strains 13.9% (range 0–29). Only three studies reported mixed GI/GII infections. The studies reported either only capsid-based genotypes or a mixture of RNA-dependent RNA polymerase (RdRp) and capsid data. Overall NoV GII.4 was the most frequently identified genotype in Africa (509/940 typed NoV; 509/832 typed NoV GII), as well as the most widely distributed strain with reports from 14 countries across Africa (Fig 2). The second most widely reported genotype was GI.3, which was detected in 10 countries and represented 30.2% of the typed NoV GI strains (32/106) and 3.4% of the total typed NoV strains (32/940). The GII.3 genotype was widely reported (8 countries) and was the predominant genotype detected in Ethiopia in 2009. Overall GII.3 was the second most frequently characterised strain across the continent (115/834 NoV GII). Other capsid genotypes that showed a wide distribution but relatively low prevalence include GII.2, GII.6, GII.14 and GII.17 (Fig 2).

**Table 2:** Additional NoV genotyping data from African studies which did not meet the inclusion criteria for prevalence estimates or only reported genotyping data.

Country	Year	Age range of study population	Genotypes	GII.4 variants	Reference
Burkina Faso	Apr-May 2011	Symptomatic children, Asymptomatic children	GI.Pd, GI.Pb, GI.P7, GII.Pg, GII.Pe, GII.P2, GII.P8	-	[44]
Cameroon	Oct-Dec 2009	Healthy children and HIV-positive adults	GI.3, GII.4, GII.8, GII.17	New Orleans 2009, Sydney 2012	[45]
Djibouti	Sep 2002 –Feb 2004	>15 years of age, acute diarrhoea	GI.11?, GII.P9, GII.P14, GII.P17, GII.P21, GII.P7/GII.9, GII.Pd/GI.3	-	[46]
Ghana	Nov 2005 –Jan 2006	<11 years	GI.P3, GI.P5, GI.P6, GI.P7, GII.P2, GII.P3, GII.P4, GII.P21	Kaiso 2003	[47]
Central African Republic and Senegal	1976–1979	-	GI.3, GII.2, GII.3, GII.6	-	[34]
South Africa	4 years 9 months 2009–2013	≤5 years	GI.P2, GI.P3, GI.P6, GI.P7, GI.PNA, GI.Pb, GI.Pf, GII.P2, GII.P4, GII.P7, GII.P13, GII.P16, GII.P21, GII.Pc, GII.Pe, GII.Pg, GII.PNA, GI.1, GI.2, GI.3, GI.5, GI.6, GI.7, GI.NA, GII.1, GII.2, GII.3, GII.4, GII.6, GII.7, GII.10, GII.12, GII.13, GII.14, GII.16, GII.17, GII.21	Osaka 2007, New Orleans 2009, Sydney 2012, unassigned GII.4	[48]

**Fig 3:**
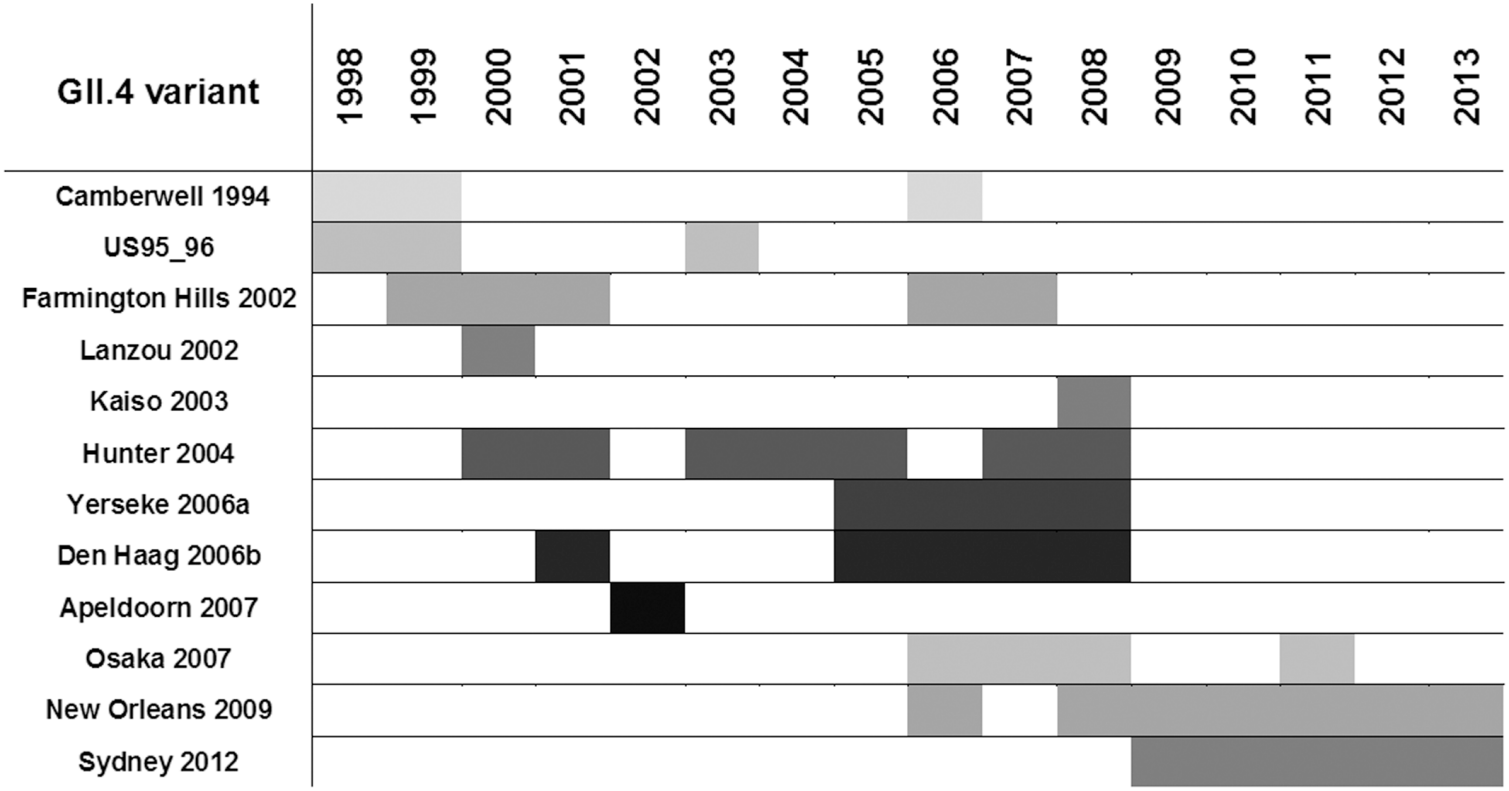
Circulation of NoV GII.4 variants between 1998 and 2013 in 12 African countries.

Between 1998 and 2013, 12 GII.4 variants were reported in NoV studies from Africa. (Tables 1 and 2, Fig 3). The early variants were all detected during the 10 year study in Malawi, which showed the circulation of major variants Hunter 2004 and Den Haag 2006b several years before they emerged as pandemic strains. The Osaka 2007 strain was detected in Ghana in the year 2000 and then reported in Egypt and Tunisia between 2006 and 2008 with its most recent detection in 2011 in SA. Interestingly, analysis of GII.4 sequences from Botswana show that the New Orleans 2009 variant already circulated there in 2006, after which it was detected in SA in 2008. The Sydney 2012 strain also circulated in parts of Africa long before its emergence, namely in Cameroon in 2009 and in Burkina Faso and SA in 2010. To date the Sydney 2012 variant has also been reported in Ethiopia, Ghana and Morocco (Tables 1 and 2). This overview of GII.4 circulation in Africa emphasises that epidemic GII.4 variants circulated in paediatric populations in Africa one to several years before their global emergence, highlighting the importance of NoV surveillance in this population. To explore whether new GII.4 variants possibly arise in Africa the first circulation of variants was compared between Africa and other countries worldwide. The Noronet database was searched using ORF2 GII.4 variant and sample date as criteria. Using this approach early circulation of Yerseke 2006a (12/2005 United Kingdom), Den Haag 2006b (Japan 2003, Slovenia 12/2005), Osaka 2007 (Netherlands 2006) and Sydney 2012 (Netherlands 2007) was identified. Since some of the GII.4 variants circulated earlier in Africa, it is possible that this continent plays a role in the emergence of novel GII.4 variants. However, more comprehensive and on-going global NoV surveillance data is needed to address this question. Apart from the GII.4 variants that could be assigned by the online NoV Genotyping Tool, there are a large number of GII.4 sequences that cannot be assigned to a variant type, based on the nucleotide sequence of the 5’-end of the capsid gene. Therefore more complete capsid gene sequences or sequences spanning the variable capsid P2 domain are needed to obtain a better understanding of GII.4 strains in Africa.

The emergence of a novel GII.17 strain in China, which replaced NoV GII.4 as the predominant cause in outbreaks of NoV gastroenteritis in 2014/2015 [49], emphasises that NoVs are dynamic and rapidly evolving and that predominant genotypes might change unexpectedly. The novel GII.17 strain was first detected in river water in Kenya in 2013 [50], where it was found to be more prevalent than GII.4, an unusual scenario, since GII.4 often represents >50% of environmental NoV-positive samples [51]. The original GII.17 strains have been detected in various African countries in sporadic cases of gastroenteritis, but more recently the novel GII.17 was reported in Morocco [29]. Due to the lack of NoV surveillance in Africa, it is likely that the novel GII.17 is circulating more widely than it is being reported, and may well emerge as the next predominant strain.

Since relatively few studies from Africa have reported combined RdRp/capsid typing data, information on NoV recombinants and their prevalence is lacking. A study in SA between 2009 and 2013 identified several recombinants which had been described in other parts of the world as early as 1999 (GII.P21/GII.3), or more recently between 2008 and 2010 (GII.Pg/GII.12, GII.Pg/GII.1), showing the global nature of NoV strain circulation. Furthermore, some novel recombinants (GII.P16/GII.17) were identified in SA which have not been described elsewhere [52]. These results highlight the need to establish not only a more continent wide and standardised approach to NoV surveillance, but the need to genotype the strains identified.

## Noroviruses in the African Environment

The prevalence and diversity of NoVs in the African environment has been investigated in surface water, sewage influent and effluent as well as shellfish (Table 3). All studies indicate significant levels of NoV contamination in treated sewage effluent and river water. Studies on surface water in Kenya and South Africa detected NoVs (GI and/or GII) in 63% of samples. The 10 most prevalent NoV genotypes detected overall in Africa in the clinical setting (Fig 2) were detected in rivers in SA and Kenya. Therefore environmental surveillance does provide genotype information on clinically relevant strains and specifically GII.4 variants. The utility of environmental NoV surveillance is further emphasised by the identification of the novel GII.17 strain in river water in Kenya [50].

**Table 3:** Prevalence and diversity of NoV in environmental samples from Africa.

Country	Year	Type of environmental sample	Prevalence of NoV %(NoV^+^/total)	Genotypes	GII.4 variants	Reference
Tunisia	Jan 2003-Apr 2007	Sewage influent and effluent Shellfish	4.4% (11/250 sewage) 1.7% (1/60 shellfish)	GI.2, GI.5, GI.9, GII.4, GI.2	Hunter 2004	[53]
Egypt	Apr 2006-Feb 2007	Sewage influent and effluent	18% (13/72)	GI.1, GI.2, GI.3, GI.5, GI.P21, GII.4*	-	[54]
Tunisia	2007–2010	Sewage influent and effluent	37% (192/518)	GI.1, GI.2, GI.4, GI.5, GI.NA, GII.12	-	[55]
South Africa	Jan 2008-Dec 2010	Surface water	63% (95/151)	GI.1, GI.2, GI.3, GI.4, GI.5, GI.8, GII.1, GII.2, GII.3, GII.4, GII.6, GII.7, GII.9, GII.12, GII.13, GII.17	New Orleans 2009, Sydney 2012, unassigned GII.4	[51]
South Africa	Aug 2010-Dec 2011	Sewage effluent	69% (35/51)	GI.1, GI.3, GI.4, GI.8, GI.NA, GII.2, GII.3, GII.4, GII.6, GII.7, GII.12, GII.13, GII.17	Osaka 2007, New Orleans 2009, unassigned GII.4	[56]
Kenya	Feb 2012-Jan 2013	Surface water	63% (25/40)	GI.1, GI.3, GI.9, GII.4, GII.6, GII.12, GII.16, GII.17	Sydney 2012, unassigned GII.4	[50]

*Nucleotide sequence not available in GenBank

## Conclusion

Despite the apparent high NoV detection rates in asymptomatic controls, NoVs are a significant cause of gastroenteritis in children in Africa. Thirty genotypes and twelve GII.4 variants have circulated between 1976–1979 and 1998–2013. Remarkable genotype diversity was observed, even in studies with a small number of NoV-positives. Although there is no NoV data for the majority of African countries the studies reviewed here have provided valuable data on NoVs in a developing and low-income setting. However, there is very little data on NoV infections in adults or the elderly. There are an estimated 25.8 million adults and children living with HIV in Sub Saharan Africa [57]. Whether this setting of a high proportion of individuals with impaired immune responses affects NoV evolution and diversity needs to be investigated. Few studies attempt to genotype both the RdRp and capsid regions, and more data on the variable domain of the capsid is needed. With the advances and relative standardisation in NoV detection and genotyping assays and enhanced access to molecular biology instruments and reagents it may become feasible to establish NoV surveillance across Africa. Utilisation of the African Rotavirus Surveillance Network to implement NoV surveillance has been suggested almost ten years ago [23] and this should be given serious consideration and support.
